# Insights into the epigenomic landscape of the human malaria vector *Anopheles gambiae*

**DOI:** 10.3389/fgene.2014.00277

**Published:** 2014-08-15

**Authors:** Elena Gómez-Díaz, Ana Rivero, Fabrice Chandre, Victor G. Corces

**Affiliations:** ^1^Department of Biology, Emory UniversityAtlanta, GA, USA; ^2^Maladies Infectieuses et Vecteurs: Écologie, Génétique, Évolution et Contrôle (UM1-UM2-CNRS 5290-IRD 224), Centre IRDMontpellier, France

**Keywords:** H3K27ac, H3K27me3, histone post-translational modifications, chromatin immunoprecipitation, gene expression regulation, mosquito-borne diseases, ChIP-seq, RNA-Seq

## Abstract

The epigenome of the human malaria vector *Anopheles gambiae* was characterized in midgut cells by mapping the distribution and levels of two post-translational histone modifications, H3K27ac and H3K27me3. These histone profiles were then correlated with levels of gene expression obtained by RNA-seq. Analysis of the transcriptome of *A. gambiae* midguts and salivary glands led to the discovery of 13,898 new transcripts not present in the most recent genome assembly. A subset of these transcripts is differentially expressed between midgut and salivary glands. The enrichment profiles of H3K27ac and H3K27me3 are mutually exclusive and associate with high and low levels of transcription, respectively. This distribution agrees with previous findings in *Drosophila* showing association of these two histone modifications with either active or inactive transcriptional states, including Polycomb-associated domains in silenced genes. This study provides a mosquito epigenomics platform for future comparative studies in other mosquito species, opening future investigations into the role of epigenetic processes in vector-borne systems of medical and economic importance.

## Introduction

Host-parasite interactions are among the most plastic and dynamic systems in nature. In these systems, epigenetic modifications can provide an accessory source of fast-acting, reversible and readily available phenotypic variation that can be directly shaped by both host and parasite selection pressures (Bonduriansky and Day, 2009; Gómez-Díaz et al., 2012). Mosquitoes are important disease vectors worldwide. *Anopheles gambiae* is major vector of malaria in Africa, a disease that affects more than 300 million people and causes around 650,000 deaths each year (Who, 2013). The genome of *A. gambiae* was sequenced more than 10 years ago (Holt et al., 2002). Since then, the roadmap for malaria control strategies has been mostly centered on the study of vector genetic variation (Severson and Behura, 2012). Yet, genetic mechanisms alone are not sufficient to explain natural phenotypic variation in terms of vector competence (Lambrechts, 2010). Thus, it is critical to expand our current understanding of mosquito-parasite interactions into an integrated view that includes both genetic and epigenetic dimensions. Although a great deal of progress has been made in deciphering the epigenetic code of *Plasmodium* parasites (see Hoeijmakers et al., 2012 for a review), knowledge of epigenetic processes in mosquitoes is limited.

Post-translational modification (PTM) of histones (acetylation, methylation, phosphorylation, sumoylation, and ubiquitinylation) is an important regulatory mechanism of transcriptional control that acts by altering chromatin structure (Kouzarides, 2007; Bonasio et al., 2010). In *Drosophila*, epigenomic profiling of enrichment or depletion patterns for specific histone modifications has been established genome-wide and this information has been correlated with the location of regulatory elements and functional domains (Kharchenko et al., 2011; Negre et al., 2011). For example H3K4me3 and H3K27ac are associated with active promoters, H3K4me1 and H3K27ac are present in active enhancers, and H3K27me3 associates with Polycomb-mediated silencing. Surprisingly, no information is available in mosquitoes on histone PTMs or regulatory elements involved in chromatin-based epigenetic processes, such as the Polycomb and trithorax complexes, insulators, and enhancers.

Available technologies now allow the analysis of whole epigenomes in a straightforward and cost-effective manner. Chromatin immunoprecipitation followed by sequencing (ChIP-seq) allows the mapping of protein-DNA interactions genome-wide. In this method DNA-protein complexes containing a specific protein of interest are immunoprecipitated from cross-linked, sonicated chromatin. DNA is purified from the enriched pool and used to generate a library for subsequent whole genome sequencing. The sequence reads for the enriched DNA are computationally aligned to the reference genome to define sharp peaks or broad blocks of modified histones. Since its development in 2007 (Robertson et al., 2007), ChIP-seq has been extensively used to survey the genomic profiles of histones and their modifications, transcription factors (TFs), DNA and histone modifying enzymes, the transcriptional machinery, and other chromatin associated proteins in various model organisms, including the fruit fly *Drosophila melanogaster* (Roy et al., 2010). One downside of this technique is, however, that its application in non-model species is still limited by the availability of reference genomes. On the other hand, in cases where the annotation of the genome is still incomplete, such as *A. gambiae*, the combination of histone modification maps and gene expression information constitutes a powerful approach that allows the functional interpretation of chromatin marks as well as *de novo* prediction of regulatory elements, genes and splice variants (Ernst and Kellis, 2010; Cheng et al., 2011). Previous studies have analyzed the transcriptome of *A. gambiae* using microarrays (Dana et al., 2005; Marinotti et al., 2005; Koutsos et al., 2007; Baker et al., 2011; Maccallum et al., 2011), but this approach gives only semi-quantitative results due to the small dynamic range of the microarray signal. A few studies have applied RNA-seq in this species on whole bodies or chemosensory appendages (Pitts et al., 2011; Vannini et al., 2014). To our knowledge no study has specifically targeted RNA-seq analyses to tissues such as the midgut or salivary glands that play a key role in the development of *Plasmodium* parasites within the mosquito, and are thus directly implicated in malaria transmission.

Using the technical and theoretical knowledge accumulated from chromatin studies in *Drosophila*, here we carry out an analysis of the transcriptome and histone PTMs in the human malaria vector *A. gambiae*. We characterize the distribution and levels of H3K27ac and H3K27me3, two key histone modifications that have been shown to play an important role in gene regulation, by performing ChIP-seq analyses in the midguts of adult mosquito females. The midgut is key tissue for *Plasmodium* development because the obligate passage of the parasite through this tissue results in large losses in parasite numbers, which may explain the frequent failure of the parasite to complete its life cycle in the mosquito (Blandin et al., 2008). We then correlate the histone profiles with levels of genome-wide gene expression obtained by RNA-seq, in order to infer functional states and predict putative regulatory elements in the mosquito. This integrative analysis allowed us to link enrichment or depletion of active and repressive histone modifications to their target genes. The result is the first platform for mosquito comparative epigenomics that can serve as a basis for future studies on the biology of mosquitoes and mosquito-borne diseases.

## Experimental procedures

### Mosquito rearing and dissection

Experiments were performed using an isogenic strain of *A. gambiae* (strain Kisumu) maintained at the MIVEGEC insectarium under standard rearing conditions (27 ± 1°C, 70 ± 10% RH and 16L: 8D photoperiod). Blood- fed adult females were allowed to lay eggs. On the day of hatching, larvae were seeded into plastic trays (25 × 35 × 7 cm) containing one liter of mineral water at a density of 300 individuals per tray. Larvae were provided with 200 mg of TetraMin® fish flakes the day after hatching, and, from then on, 400 mg TetraMin every 2 days until pupation. On pupation, trays were placed inside an emergence cage (27 × 40 × 35 cm) and provided with an *ad libitum* source of 10% sugar solution for the emerged adults. Dissection of the midguts and the salivary glands was performed on adult females aged 6–8 days. Tissues were maintained in ice-cold Schneider's insect culture medium (Sigma-Aldrich) to avoid degradation, and fresh tissues were immediately processed for chromatin and RNA analyses.

### RNA isolation and RNA-seq library preparation

Total RNA was extracted from fresh mosquito tissues (~25 midguts and ~ 50 salivary glands) using the *mir*Vana™ RNA Isolation Kit (Ambion®) according to the manufacturer's protocol. RNA concentration was quantified using a Qubit® 2.0 Fluorometer, and RNA integrity was determined with an Agilent 2100 Bioanalyzer. Illumina libraries were prepared and sequenced at the HudsonAlpha Institute for Biotechnology, using an Illumina HiSeq2000 sequencer. Standard directional RNA-seq library construction, 50 bp paired end reads with ribosomal reduction (RiboMinus™ Eukaryote Kit, Ambion®).

### Chromatin isolation, immunoprecipitation, and sequencing

Antibodies to histone modifications H3K27ac (Abcam ab4729) and H3K27me3 (Millipore 07-449) used in this study have been previously tested and shown to specifically recognize the appropriate epitopes (Kharchenko et al., 2011; Landt et al., 2012). The epitope sequences are conserved between *Drosophila* and *A. gambiae*.

For immunoprecipitation experiments, mosquito tissues (15–20 midguts) were suspended in 100 μl of Schneider's medium, fixed by adding 37% formaldehyde to a final concentration of 1%, and incubated at room temperature for 10 min. The reaction was stopped by adding 1/10 volumes of 1.25 M glycine solution, incubated for 5 min before centrifugation at 4000 rpm for 3 min, and washed twice using cold 1X PBS. Tissues were lysed in 200 μl of cell lysis buffer (5 mM PIPES pH 8.0, 85 mM KCl, 0.5% NP40), and protease inhibitor cocktail (complete protease inhibitor cocktail tablets, Roche), then homogenized with a pestle mixer and incubated on ice for 15 min. After centrifugation at 4000 rpm for 8 min, the nuclei were resuspended in 200 μl of nuclear lysis buffer (50 mM Tris-HCl pH 8.0, 10 mM EDTA.Na2, 1% SDS, and protease inhibitors), and incubated on ice for 20 min. Then, 100 μl of IP dilution buffer (0.01% SDS, 1.1% Trition X-100, 1.2 mM EDTA.Na2, 16.7 mM Tris-HCl pH 8.0, 167 mM NaCl) were added. Sonication was performed using a Diagenode BioRuptor using 40 cycles of 30 s ON/ 30 s OFF, resulting in fragments around 300–500 bp, followed by centrifugation at 4°C for 10 min. Quality control of fixed chromatin was performed by visualization of seared fragments by agarose electrophoresis prior to proceeding with the chromatin IP. For the preclear step, 50 μl of protein A magnetic beads (Dynabeads® Protein A, Novex®) were pre-coated using anti-rabbit IgG in 5% BSA. Beads were then added to each sample diluted 5X in IP dilution buffer, and incubated overnight (OVN) at 4°C in a rocker. Seventy five μl (5%) were taken as a control input from this solution and subjected to the same experimental procedure than the test sample, except for the antibody-binding step. For the test sample, 3 μl of Ab (1 μg/μl) pre-bound to 50 μl of protein A beads were added to the pre-cleared chromatin and incubated OVN at 4°C. After discarding the supernatant by magnetic separation, antibody-bound chromatin was subjected to 3 washes in low salt buffer (0.1% SDS, 1% Trition X-100, 2 mM EDTA.Na2 pH 8.0, 20 mM Tris-HCl pH 8.0, 150 mM NaCl), 2 washes in high salt buffer (0.1% SDS, 1% Trition X-100, 2 mM EDTA.Na2 pH 8.0, 20 mM Tris-HCl pH 8.0, 500 mM NaCl), 2 washes in LiCl buffer (1 mM EDTA.Na2 pH 8.0, 10 mM Tris-HCl pH 8.0, 0.25 M LiCl, 1% NP40, 1% SDS), and one final wash in TE. Elutions were then performed by adding 2 × 200 μl of IP Elution Buffer (0.1 M NaHCO_3_, 1% SDS). Crosslinks were reversed by incubating the ligated chromatin at 65°C OVN with the addition of 0.25 M NaCl, 10 mM EDTA, and 40 mM Tris. Extraction was performed by adding 8 μl of proteinase K (10 mg/ml) and incubation at 50°C for 2 h. The DNA was extracted following a standard phenol-chloroform procedure.

Sequencing libraries were prepared following the protocol described by Bowman et al. (2013) optimized for low quantity DNA. Before library preparation, ChIP DNA was pre-cleaned using a 1.8 × ratio of SPRI beads (Agencourt AMPure XP, Beckman Coulter). End preparation was performed by end repair (End-It DNA End Repair Kit, Epicenter Cat# ER0720) and addition of “A” base to 3′ends (Klenow 3′-5′exo, NEB Cat# M0212S). Adaptor ligation was performed using a universal adaptor sequence and T4 ligase (NEB Cat# M0202S), and the ligated products were SPRI cleaned (1.6 × beads ratio). Amplification of ChIP libraries was performed by qPCR using an Applied Biosystems 7500 Fast Real-Time PCR System. Reactions (50 μl) consisted of 0.2 μM universal primer, 0.2 μM barcoded primer and 25 μl of KAPA SYBR FAST qPCR Master Mix (KapaBiosystems). Real time kinetics was monitored and library amplifications were stopped at the end of the extension after SYBR green reported reaction kinetics in the log phase (usually 15 cycles). A final SPRI purification with 1.0 × beads ratio was performed on the ChIP DNA libraries to remove adapter/primer dimers (120 bp) from the rest of the library (250 bp and above). ChIP libraries were sequenced at the HudsonAlpha Institute for Biotechnology using an Illumina HiSeq2000 sequencer.

### Data analyses

#### RNA-seq data analysis

RNA raw sequencing data were quality checked using FastQC, and trimmed on both ends, based on the quality estimates for each sequence, by using the FASTX-Toolkit (http://hannonlab.cshl.edu/fastx_toolkit). For the analysis of RNA paired directional reads we used the Tuxedo suite of programs as described by Trapnell et al. (2012). First, TopHat v2.0.10 (Trapnell et al., 2009) was used to map reads to the GTF annotation file of the most recent version of the *A. gambiae* genome, assembly AgamP4 PEST strain, available at VectorBase (https://www.vectorbase.org/). Reads were aligned using default parameters, except the option of library type set as first-strand for directional RNA-seq, and the inner mate distance parameter r, which was estimated using the CollectInsertSizeMetrics tool implemented in the Picard's tools suite (http://picard.sourceforge.net/index.shtml). The Cufflinks v2.1.1 package (Roberts et al., 2011), which includes Cuffcompare, Cuffmerge and Cuffdiff; was used to quantify transcript abundance in terms of Fragment Per Kilobase Million (FPKM), allowing the discovery of new transcripts. Parameters were set as default using the GTF option, bias correction, and applying the total hits norm. We then compared the new transcriptomes to existing annotated transcripts using Cuffcompare. We ran Cuffdiff first on the conserved, known transcripts, and then on the combined transcriptome, which includes both previously annotated and new transcripts, to examine tissue specific differences in gene expression between midguts and salivary glands. The clusters of differently expressed genes were visualized using the R/Bioconductor cummeRbund package (http://compbio.mit.edu/cummeRbund/). Significant expression differences between tissues was defined as having both a *P*-value < 0.01 and a false discovery rate (FDR) of <0.05.

#### Chip-seq data analyses

Quality metrics statistics of Illumina reads were obtained using FastQC (http://www.bioinformatics.bbsrc.ac.uk/projects/fastqc). We used Bowtie v.1.0.0 (Langmead and Salzberg, 2012) to map reads from control and ChIP libraries to a custom genome index for *A. gambiae*; built using the most recent AgamP4 reference assembly using the bowtie-*build* tool. We ran Bowtie using the—m option to filter repetitive sequences. To identify significantly enriched regions for H3K27ac we performed peak calling analysis using MACS v1.4.2 (Zhang et al., 2008; Feng et al., 2012). Parameters were set to consider equal numbers of unique reads for input and ChIP samples, remove redundant reads (PCR duplicates), and a *p*-value cutoff of 1 × 10e-5. To analyze the distribution of H3K27me3 we used SICER, which is optimized for the analysis of broad ChIP-enrichment regions (Zang et al., 2009; Xu et al., 2014) characteristic for this histone modification mark. Parameters were set to a window size of 200, gap size 600 and FDR cutoff of 1 × 10e-5, and the default value for the redundant rate cutoff.

We used BEDTools2.19.0 (Quinlan and Hall, 2010) to obtain genome coverage for each histone modification mark, which is the percentage of the genome in bp that shows significant enrichment of H3K27ac and H3K27me3, as well as the intersects of H3K27ac and H3K27me3 enrichment peaks with various genomic features (promoters, TSSs and genes). For these analyses we define promoters as regions located within a distance of 200 bp upstream and downstream of the transcription start site (TSS).

To examine the association between H3K27ac and H3K27me3 profiles genome-wide, we first normalized control and ChIP samples based on read counts and calculated the difference using DeepTools (Ramírez et al., in press). The resulting enrichment files were binned into 10 bp windows covering the entire *A. gambiae* genome reference sequence. We then referenced the 10 bin normalized and subtracted ChIP enrichments to gene coordinates of the AgamP4 gene set and calculated average enrichments per gene across a region that includes the gene body scaled to 500 bp, flanked by 200 bp before the TSS and after the Transcription Termination Site (TTS), respectively. The same type of analysis was performed by referencing ChIP data to gene coordinates from the “custom” gene set generated by CuffCompare, which merges novel and conserved transcripts (see above). We then applied K-means clustering to the matrix of enrichment values for H3K27me3 to organize genes based on their similarities into 3 clusters using Cluster v3.0. Genes within each cluster were ordered by decreasing mean enrichment values. Groups of H3K27me3 enrichment were then used as anchors to order the matrix of mean values of H3K27ac and gene expression. Clustered data was then visualized as a heatmap using deepTools.

Combined analyses of ChIP and RNA-seq data were performed in R 3.1.0 (http://www.r-project.org/) using Bioconductor (http://www.bioconductor.org). We used BedTools v2.19.0 and SamTools v1.4 (http://samtools.sourceforge.net) for file manipulation and conversion. Coverage analyses on bed and bam files were performed using BedTools and DeepTools suites.

#### Functional analysis of genes

Functional analyses were performed on genes obtained from the clustering approach corresponding to the various enrichment profiles (see results), and that intersect with H3K27ac and H3K27me3 peaks identified by MACS or SICER. Sequences for the various gene sets were retrieved by mapping VectorBase gene IDs against the UniProtKB database (http://beta.uniprot.org/). We employed Blast2Go (http://www.blast2go.de/) to conduct Gene Ontology (GO) and KEGG (Kyoto Encyclopedia of Genes and Genomes) pathways annotations. Fisher's exact test was used to retrieve significantly enriched GO terms for genes marked with H3K27ac and H3K27me3 using Blast2Go. GO functional categories are defined as those containing at least five genes and a minimum enrichment score of 1.3 (*p*-value < 0.05).

### Real-time PCR validation

Validation by ChIP-qPCR was performed by targeting two housekeeping genes, actin5C and TER94, which typically show significant enrichment of H3K27ac in *Drosophila* (http://www.modencode.org/). For this assay we also targeted an intergenic region located 2 Kb upstream of these two genes as a negative control. Reactions were performed using SYBR Green I Master kit (Applied Biosystems) in an Applied Biosystems Lightcycler. The PCR parameters are: 1 cycle of 10 min at 95°C, 40 cycles of 10 s at 95°C, 10 s at 60°C, and 20 s at 72°C. PCR primer sequences are listed in Table S1.

## Results

### Gene expression and transcript discovery

RNA library processing and quality metrics are shown in Table 1. The expression analysis on the assembly of reads obtained from *A. gambiae* female mosquito tissues, midguts and salivary glands, resulted in a total of 13,969 novel transcripts that correspond to newly discovered non-annotated loci (class code “u”: Unknown, intergenic transcript); combined GTF and BED files available at GEO GSE59773) as well as transcripts categorized as class code “=”correspond in most cases to extensions of previously annotated genes based on intron matches. Most novel transcripts (6907 in midguts and 6943 in salivary glands) show relative abundances, expressed as Fragments Per Kilobase of exon model per Million mapped fragments (FPKM), above the 95 percentile of expression for the distribution of transcript abundances, which we estimated correspond to a value of 0.26 for midguts and 0.56 for salivary glands (lower bound of the 95% confidence interval (CI) for the distribution of transcript abundance). Thus transcripts that show FPKM values above this threshold can be considered as reliable in terms of novel transcript discovery and gene expression differences. Blasting a subset of these transcripts against non-redundant protein databases confirms that they are novel (data not shown). This supports the notion that there is a large number of genes in the reference genome of *A. gambiae* that remain to be discovered and/or properly annotated.

**Table 1:** **Statistics of ChIP-seq and RNA-seq data processing and quality checks**.

	**No. reads**	**Reads mapped**	**Reads excluded**	**Reads analyzed**	**%GC**	**Quality score**
**ChIP-seq**						
H3K27ac	24770844	13300823 (53.7%)	3401339 (13.7%)	12286069 (49.6%)	45	38
H3K27me3	22053926	12832729 (58.2%)	3861626 (17.5%)	10249493 (46.5%)	49	38
Input	19456294	9684252 (49.8%)	3235677 (16.6%)	9068488 (50.7%)	42	38
**RNA-seq**						
Midgut	83046282	60149597 (72.4%)			52	37
Salivary gland	78884200	57346015 (72.7%)			52	37

**Figure 1:**
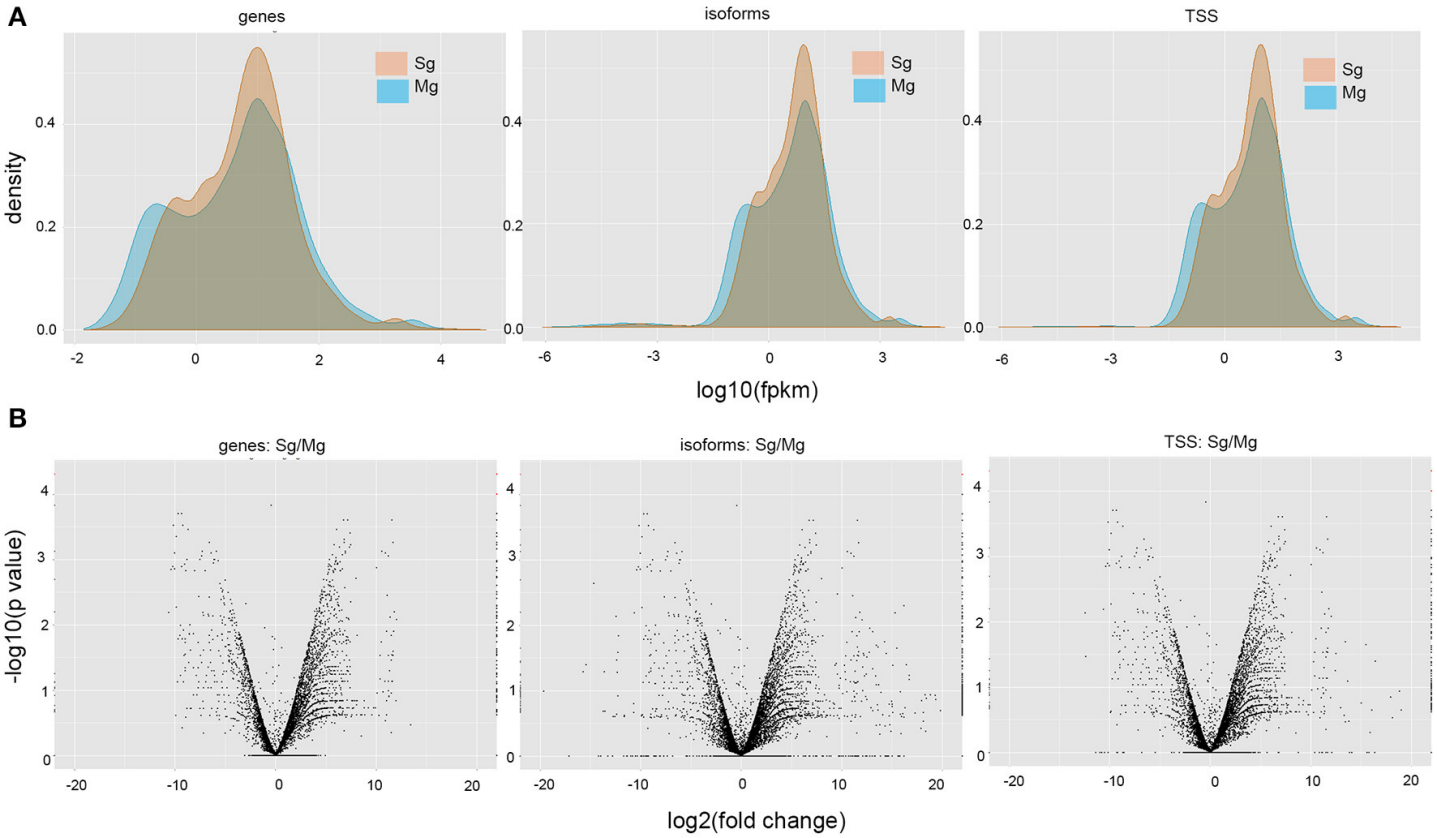
**Differential expression analyses between midgut and salivary gland samples of *A. gambiae*. (A)** Volcano plots displaying changes in gene expression, isoforms, and TSS usage (log2-fold change). Each symbol represents one gene that has detectable expression in either tissue. Relative differences in signal intensity along the X-axis reflect up-regulation in salivary glands when positive and up-regulation in midguts when negative. The Y-axis displays log10-transformed *p*-values associated with tests of differential gene expression; red symbols indicate significant values for a threshold level of 0.05. **(B)** Density plots showing the expression level distribution for all genes, isoforms, and TSS, in the two tissues. FPKM, fragments per kb of transcript per million fragments mapped.

The comparison between transcripts obtained from midguts and salivary glands identified a total of 23 differentially expressed genes, 23 TSS and 21 isoforms switching events (*p*-value < 0.01, *q*-value < 0.05) (Figure 1). We did not detect significant differences in expression when considering the combined transcriptome, which includes both previously annotated and novel genes (Figure 1), probably due to low abundances of novel transcripts, which represent half of the combined data set, and which may have bias quantification results. Note that these are conservative estimates based on the threshold used by Cufflinks.

### Validation of the ChiP-seq approach and mapping of H3K27ac and H3K27me3 in *A. gambiae*

Statistics of data processing are presented in Table 1. ChIP and control libraries passed all quality tests (Figure S1). The percentage of reads mapped to the mosquito genome was 53.70% for H3K27ac (13,300,823 reads) and 58.19% for H3K27me3 (12,832,729 reads). Of these, 13.73% for H3K27ac and 17.51% for H3K27me3 reads were identified by Bowtie as corresponding to repetitive sequences such as those typically found in transposons and centromeric or telomeric regions. The percentage of mapping is similar between the two target histones and the control. Results of the ChIP-qPCR validation are also consistent with data obtained by the ChIP-seq analysis (data not shown). Taken together, these results indicate that the ChIP-seq data provide an accurate representation of the genome-wide distribution of histone modifications analyzed in *A. gambiae* and offer proof-of-concept validation of the approach.

Coverage analyses of ChIP peaks regions reveal that 6.4% of the genome is occupied by H3K27ac, whereas 22% is occupied by H3K27me3. The fraction of the genome covered by each histone modification across different chromosomes is shown in Table 2. By applying a peak calling approach using either MACS or SICER we identified 6639 peaks for H3K27ac and 12,939 peaks for H3K27me3 after input control normalization. Of the peaks identified for H3K27ac, 70.2% (4657) intersect with annotated genes or their promoters (here defined as regions located 200 bp upstream and downstream of TSSs), whereas a small fraction of the remaining peaks (29.8% of total) correspond to intergenic regions. In the case of H3K27me3, only 41% of the peaks (5414) intersect genes or their promoters and, therefore, most peaks appear to be intergenic. When considering the custom gene set that includes the newly discovered transcripts, the percentage of peaks that overlap to genes is 6156 for H3K27ac and 7813 for H3K27me3, thus indicating that a considerable proportion of ChIP signal maps to non-annotated regions.

**Table 2:** **Coverage of H3K27ac and H3K27me in the *A. gambiae* genome by chromosome**.

**Chromosome**	**H3K27ac**		**H3K27me3**		**Size (bp)**
	**Read depth**	**% cov**	**Read depth**	**% cov**	
2L	4051943	8.21	10503751	21.28	49364325
2R	5094054	8.28	15188866	24.68	61545105
3L	2984278	7.11	8646592	20.61	41963435
3R	3645201	6.85	11609840	21.82	53200684
UNKN	111428	0.26	249640	0.59	42389979
X	1661196	6.81	6941572	28.45	24393108
Genome	17548100	6.43	53140261	19.45	273109044

*The depth of coverage in bp, the fraction of the chromosome covered, and the total size in bp for each chromosome and the complete genome is indicated*.

### Genome-wide profiling of histone and gene transcriptional states

**Figure 2:**
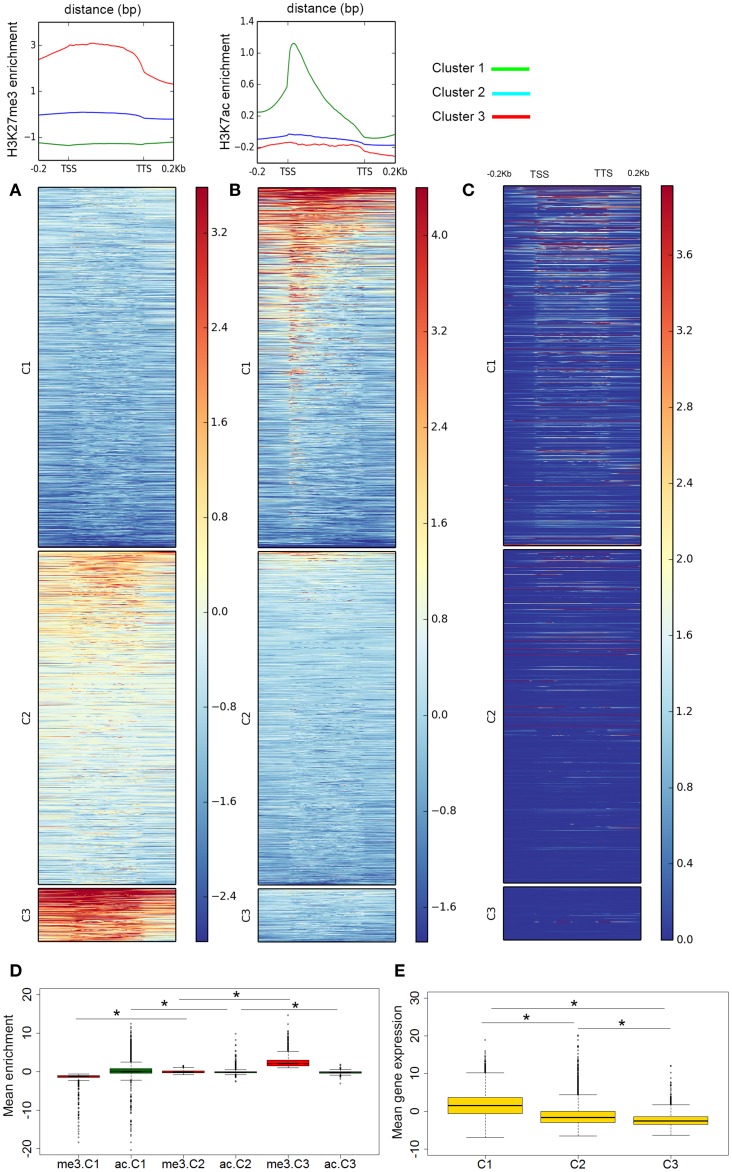
**Genome-wide distribution of histone modifications**. Distribution of **(A)** H3K27me3 and **(B)** H3K27ac with respect to gene features in *A. gambiae* midguts. The enrichment or depletion is shown relative to chromatin input. The regions in the map comprise gene bodies flanked by a segment of 200 bp at the 5′ end of TSSs and TTSs. Average profiles across gene regions ±200 bp for each histone modification are shown on top. **(C)** Heatmap of RNA-seq data showing the level gene expression, as read count, profiled along the region. In all heatmaps **(A–C)** genes were organized into 3 clusters based on their level of H3K27me3. For H3K27ac, cluster genes are ordered by descent but independent to H3K27me3. The color bars indicate the range of intensities based on ChIP enrichment, from red to blues for higher to low enrichment values. Boxplots showing the mean enrichment of H3K27ac and H3K27me3 by cluster **(D)**, and mean level of gene expression by gene cluster **(E)**. Significant pairwise comparisons are indicated by asterisks (see text).

To examine the patterns of histone acetylation and methylation genome-wide, we considered *A. gambiae* genes plus flanking regions of 200 bp upstream and downstream of the TSS and TTS, respectively. The distribution maps of the two histone modifications at genes revealed a mutually exclusive pattern of H3K27ac and H3K27me3 enrichment genome-wide (Figure 2). The same pattern was obtained when using the standard annotated AgamP4 or our custom gene-set as anchors. When examining the average histone modification profiles across the gene regions, results show peak maxima of H3K27ac downstream of the TSSs of genes (Figure 2B), whereas H3K27me3 tends to occupy broader regions that cover the entire body of genes (Figure 2A). K-means clustering of ChIP-seq data resulted in the classification of genomic regions into three clusters based on their H3K27me3 mean enrichment profiles. Cluster 1 genes, (C1: 12,913 genes) are depleted of H3K27me3 and contain high levels of H3K27ac. This cluster may correspond to genes that are highly transcribed. Cluster 3 genes (C3: 2021 genes) contain high levels of H3K27me3 and they are depleted of H3K27ac. Genes in this cluster probably correspond to Polycomb-silenced genes in *Drosophila*. Interestingly, a third gene cluster identified based on H3K27me3 levels, cluster 2 (C2: 11,976 genes) contains intermediate levels of H3K27me3 and are also generally depleted of H3K27ac but not as dramatically as genes in C3 (Figure 2D). Pairwise comparisons between clusters revealed significant differences in mean enrichment values for genes marked with H3K27ac (C1 = 0.320 ± 0.013, C2 = −0.100 ± 0.004, C3 = −0.198 ± 0.007; Kruskal-Wallis, all *p*-values < 0.001), or H3K27me3 (C1 = −1.281 ± 0.005, C2 = −0.017 ± 0.004, C3 = 2.525 ± 0.033; Kruskal-Wallis, all *p*-values < 0.001; Mean values of ChIP enrichment by gene, normalized and input subtracted). It is also noticeable from the heatmaps that at regions where H3K27me3 localize at high levels, the signal often expands toward the 3 and 5′ end of the genes covering most of the flanking region. Likewise, a closer look at genes marked with H3K27ac reveals two sets of genes based on the distribution across the region: genes that have this histone modification at the promoter and coding region and a second group of intermediate enrichment where this mark is centered on promoters (Figure 2B).

We next asked how these various histone modification profiles relate to the transcriptional status of genes. Considering genes, annotated and novel, that intersect with H3K27ac and H3K27me peaks, we found significant differences in gene expression between genes containing each of these two histone modification (Kruskal-Wallis chi-squared = 7138.69, *df* = 1, *p*-value = 0). Comparison of histone modification maps and gene expression genome-wide reveals a preferential localization of H3K27ac in active genes and promoters whereas genes that are marked with H3K27me3 are silent or are transcribed at low levels (Figure 2C). Indeed, this gene expression pattern is also true in quantitative terms when considering the gene clusters defined based on the levels of enrichment or depletion of histone PTMs (C1: 1.60 ± 0.029, C2: −1.22 ± 0.03, C3: −2.18 ± 0.05; Kruskal-Wallis chi-squared C1–C2 = 4646.224, *df* = 1; C2–C3 = 238.38, *df* = 1; C1–C3 = 2097.31, *df* = 1, all *p*-values < 0.001; Mean gene expression by gene are calculated based on the log2 of FPKM values) (Figure 2E).

### Functional annotation of histone modification profiles

**Figure 3:**
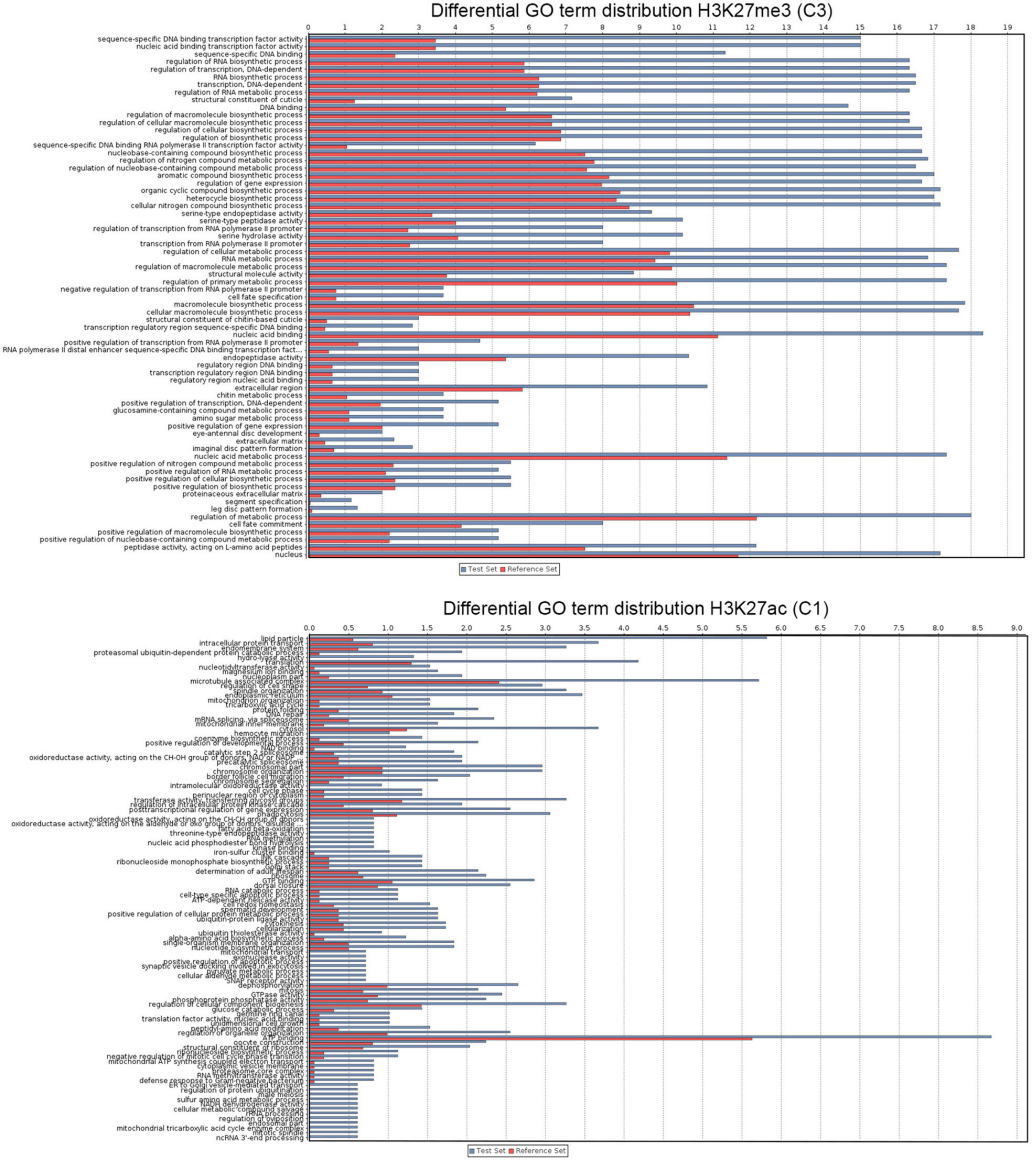
**Functional analysis of *A. gambiae* genes**. The graphs show GO terms significantly associated with genes that show significant enrichment or depletion of H3K27ac and H3K27me3 at high levels (see Figure 2). Bars corresponds to number of sequences associated with each GO term. In the case of genes marked with H3K27ac from cluster 1 due to the number of records only most significant GO terms are shown (see Table S2 for the complete list).

**Figure 4:**
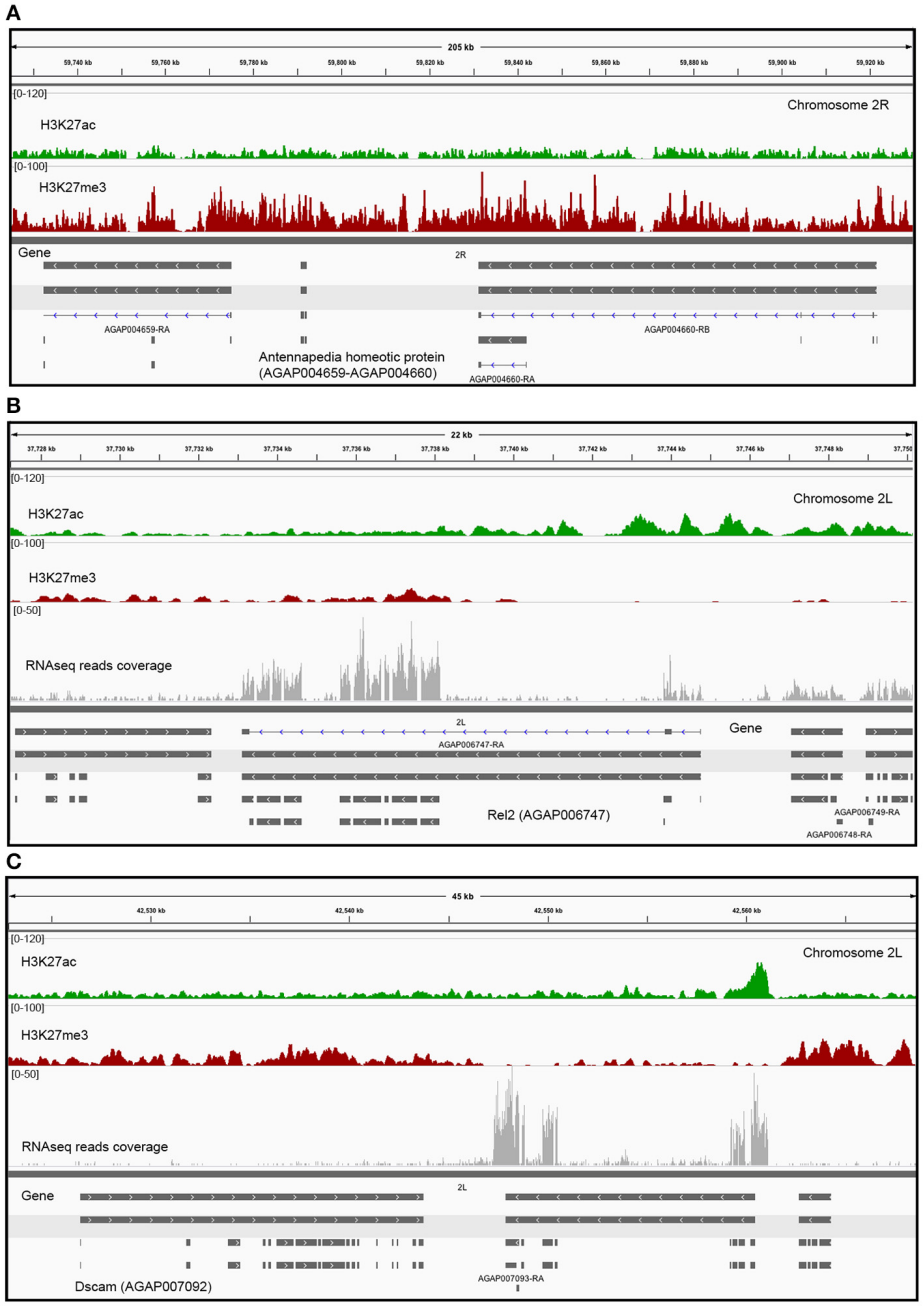
**Histone modification levels in selected genes. (A)** Enrichment profile of histone PTMs in two *Hox* genes of *A. gambiae* (Antennapedia homeotic proteins, AGAP004659-AGAP004660). These are classic examples of *Drosophila* developmental genes where H3K27me3 is highly enriched and distributed in large domains that encompass both genic and intergenic regions in cells where the *Antennapedia* gene is silenced. Examples of various histone modification profiles, based on the level of enrichment or depletion in H3K27ac or H3K27me3, in two candidate genes: **(B)** *Rel2* (AGAP006747, GO:0005515), and **(C)** *Dscam* (AGAP007092, GO: GO:0005515) (Tables S2, S3). The scale of the tracks is proportional to the number of sequencing reads for each histone modification. Gene expression in terms number of RNA reads mapped to each gene is also displayed.

Genes clustered based on their histone modification profile, either marked with H3K27ac or H3K27me3, were assigned to GO accession numbers and classified into functional categories under three major classes (biological process, cellular component, and molecular function). We found multiple GO categories associated with each cluster (Table S2). Genes enriched in either H3K27ac or H3K27me3, were assigned to multiple different KEGG pathways. Of these, pathways involved in metabolism of amino acids and signaling pathways were top ranked hits for genes marked with H3K27ac or H3K27me3 (Figure S2). The genes associated to each histone PTM (see Figure 2 for cluster names) also differ in the relative enrichment of GO terms (Figure 3, *p* < 0.05). Particularly, genes in cluster 1, highly enriched in H3K27ac, and cluster 2, containing intermediate levels of H3K27ac and H3K27me3, display a significant differential enrichment in GO terms associated with metabolic processes, immune, and signaling pathways. This is in marked contrast with genes in cluster 3, which display differential enrichment in GO terms related to membrane transporters and developmental processes (Figure 3, Table S2). One classic example of developmental genes that fall into this category and are associated with Polycomb and H3K27me3 domains in *Drosophila* are the homeobox genes (Schwartz et al., 2006) (Figure 4A, GO:0005634 Table S2). We then asked whether there is an association between the GO terms retrieved for genes differentially marked with H3K27ac and H3K27me3, and GO terms that have been linked to malaria infection and mosquito resistance to insecticides (Table S3). Interestingly, genes marked with these two histone modification marks include immunity related genes such as Toll, Immune Deficiency (IMD) and Janus kinase/signal transducers and activators of transcription (JAK/STAT) pathways; antimicrobial effectors such as defensins and cepropins; and metabolic genes such as the Vitellogenin gene. Examples of histone modification profiles of two of these candidate genes, which are significantly associated with enriched GO terms, are shown in Figure 4B, and the histone modification profiles for each candidate gene examined are shown in Table S3.

## Discussion

Changes in chromatin state play an important role in a number of biological processes, including responses to stress and environmental conditions. In organisms like *Drosophila* that lack DNA methylation, post-translational modifications of histones are an important layer of gene regulation (Bonasio et al., 2010; Bannister and Kouzarides, 2011). However, chromatin maps and the dynamics of epigenetic control in mosquitoes have remained to date unexplored. In order to fill this gap we describe genome-wide maps of two histone modifications with key roles in gene regulation, H3K27ac and H3K27me3. To establish a link with gene expression, we then correlate the presence of these modifications in specific genes with their transcriptional state in midguts and salivary glands of *A. gambiae* females.

In *Drosophila*, various combinations of active and repressive histone marks have been found to be associated with distinct chromatin functional states (Filion et al., 2010; Roy et al., 2010; Kharchenko et al., 2011; Negre et al., 2011; Yin et al., 2011). Two of these histone modifications that display contrasting distribution patterns and regulatory functions include acetylation and methylation of lysine 27 of histone 3. Previous studies have shown that H3K27ac is typically enriched at enhancers, TSSs and coding regions of active genes, whereas H3K27me3 localizes to repressed loci (Tolhuis et al., 2006; Kharchenko et al., 2011; Kellner et al., 2012; Van Bortle et al., 2012; Brown and Bachtrog, in press). Results described here for the mosquito *A. gambiae* agree with these observations and suggest a close association between the presence of H3K27ac and H3K27me3, and the transcriptional state of genes.

Genome-wide histone maps in *A. gambiae* reveal that H3K27ac and H3K27me3 cover approximately 6 and 14% of the genome, respectively. These two genome fractions do not overlap and there is a mutually exclusive pattern of enrichment at most genes: H3K27me3-containing promoters and genes show no enrichment for H3K27ac, and, reciprocally, those containing H3K27ac are not enriched for H3K27me3 (Figure 2). This is in agreement with previous studies in *Drosophila*, and supports the notion that these two histone modifications also have opposite biological functions in mosquitoes. Despite a general mutual exclusive pattern in the distribution of methylated and acetylated H3K27 sites, we found some regions of overlap, including both genic and intergenic regions. A possible explanation for this pattern would be promoter bivalency, which occurs when gene promoters contain both active and repressive modifications. These promoters are considered poised for activation (or repression) at a later stage. But this pattern has been described in the context of differentiating cells or during development (Creyghton et al., 2010), and is restricted in principle to promoters containing activating histone H3K4me3 and repressive H3K27me3 marks, not H3K27ac (Voigt et al., 2013). Alternatively, the presence of the two histone modifications can be the result of heterogeneous enrichments across cells as it has been recently been shown using single-cell ChIP-seq technologies (Wang and Bodovitz, 2010). In the case of intergenic regions, co-localization of H3K27ac and H3K27me3 has been associated with the presence of enhancer elements (Creyghton et al., 2010). To validate this hypothesis in mosquitoes it would be necessary to map other histone modifications such as H3K4me1 that are diagnostic for this type of regulatory elements (Visel et al., 2009; Zhu et al., 2013).

The combined analysis of ChIP-seq and RNA-seq data in midgut cells of *A. gambiae* supports a relationship between transcription levels and profiles of histone PTMs. We find that the presence of H3K27ac coincides with actively transcribed genes, whereas H3K27me3 is mostly associated with clusters of repressed genes that show low levels of transcription. Our analyses also suggest that enrichment or depletion of H3K27ac and H3K27me3 correlates not only with the transcriptional state of genes but also with the magnitude of their expression or silencing, which is in agreement with earlier studies in *Drosophila* (Negre et al., 2011). For example, in flies and mammals it has been shown that H3K27me3 can form domains that can expand 100 kb or more and covers blocks of silent genes and intergenic regions, and expressed genes that are preferentially located in regions immediately flanking H3K27me3 BLOCs (Pauler et al., 2009; Young et al., 2011; Hou et al., 2012). Many of these domains can also contain Polycomb (Schwartz et al., 2006; Tolhuis et al., 2006; Schuettengruber et al., 2009). In this study, Polycomb-containing genes would correspond to those of cluster 3 in Figure 2. One example of this type is the *Hox* genes of *Drosophila*, which are active in embryonic and larval stages of the life of this organism. These *Hox* genes have their homologous in mosquitoes and they display a pattern of H3K27me3 enrichment as broad peak blocks in differentiated cells of the midgut (Figure 4). It has been shown in *Drosophila* that H3K27me3 can be also be present in genes in euchromatic regions as focal peaks, rather than in domains, where there is a dynamic turnover between active and repressive histone marks and where these marks participate in the fine tuning of transcription (Riddle et al., 2011; Young et al., 2011). In the case of *A. gambiae* such a distribution pattern would correspond to genes of cluster 2, which show intermediate levels of H3K27ac and H3K27me3 and medium to low transcription (Figure 2). Finally, genes containing H3K27ac show different enrichment levels in this histone PTM. Particularly interesting is the finding of a subset of genes that have this modification only at promoters and show intermediate levels of transcription, suggesting that RNA Polymerase II may be paused at these regions (Figure 2). This has been previously reported by ChIP-seq studies in flies and mammals that mapped RNA-polymerase binding patterns in association with various histone PTMs and regulatory elements genome-wide (Negre et al., 2011; Adelman and Lis, 2012). These findings together support the conclusion that mechanisms of regulation of chromatin structure and function have been conserved between *Anopheles* and *Drosophila*.

The functional analysis of genes differentially enriched in histone PTMs further supports the idea that histone modification profiles correlate with gene expression in *A. gambiae*. This finding, and the similarities found with the mechanisms of epigenetic regulation described in *Drosophila*, has potentially important implications in the context of infection and the response of mosquitoes to environmental stresses. We further explored this issue by examining the enrichment in H3K27 PTMs and the transcriptional status of a set of target genes that have been previously shown to be up-regulated in response to malaria infection as well as genes involved in insecticide resistance (referenced in Table S3). Interestingly, the majority of the candidate genes examined appear to be enriched in one of the two histone modifications, and most fall within clusters 1 and 2, which suggests that they are in a dynamic on/off switch for transcriptional regulation (Table S3).

One limitation of epigenomic studies in non-model organisms is the availability of fully annotated genomes that allow high resolution mapping of epigenetic modifications and regulatory elements to their target genes. This is clearly illustrated in this study, and may be one of the reasons why analysis of epigenetic modifications has remained unexplored in mosquitoes. The analysis of the *A. gambiae* transcriptome in midguts and salivary glands using RNA-seq resulted in the identification of several thousand novel transcripts. The high discovery rate is not surprising considering that the majority of transcriptome studies carried out to date in *A. gambiae* have employed microarrays (Baker et al., 2011; Maccallum et al., 2011). It is important to note that a large fraction of regions enriched for H3K27ac and H3K27me3 map to non-annotated genes. The differential enrichment in histone modification marks and tissue specific signatures of transcription at these genes can be important in predicting potential targets in the response of *A. gambiae* to pathogens and environmental stressors.

## Conclusions and future directions

Here we present an integrative approach that combines genome-wide profiling of histone modifications and gene expression that allows the functional interpretation of chromatin modifications as well as *de novo* prediction of regulatory elements and genes. Results support a link between changes in histone modification profiles and transcription levels in *A. gambiae*, and this includes genes that have been previously identified as having important roles in the epidemiology and control of malaria transmission. The patterns obtained are similar to those previously described in *Drosophila*, suggesting the existence of common mechanisms of chromatin regulation in *A. gambiae*. These findings open new opportunities to study the dynamics of chromatin states including other histone PTMs in various developmental stages and environmental conditions (i.e., infection or exposure to insecticides) as well as the mechanisms by which these states are controlled by regulatory elements such as enhancers and insulators.

## Data access

ChIP-seq and RNA-seq raw data as well as processed data (wig, bed, FPKM values and GTF files) are deposited in GEO database under accession number GSE59773.

### Conflict of interest statement

The authors declare that the research was conducted in the absence of any commercial or financial relationships that could be construed as a potential conflict of interest.

## Supplementary material

Figure S1 **Fingerprint plot that shows the distribution of reads, as the cumulative sum of read counts in 10 bp window bins, for H3K27ac, H3K27me3, and input samples**. A tight diagonal is expected when reads are equally distributed across the genome, as is the case for the input, whereas the curve becomes more pronounced as the degree of enrichment increases and is more localized in the ChIPs relative to the control sample.

Figure S2 **Piecharts that show the number of sequences assigned to KEGG pathways for each histone modification gene cluster (see text, Figure 2)**.

## References

[B1] Adelman K. Lis J. T. (2012). Promoter-proximal pausing of RNA polymerase II: emerging roles in metazoans. Nat. Rev. Genet. 13, 720–731 10.1038/nrg3293 22986266 PMC3552498

[B2] Baker D. Nolan T. Fischer B. Pinder A. Crisanti A. Russell S. (2011). A comprehensive gene expression atlas of sex- and tissue-specificity in the malaria vector, Anopheles gambiae. BMC Genomics 12:296 10.1186/1471-2164-12-296 21649883 PMC3129592

[B3] Bannister A. J. Kouzarides T. (2011). Regulation of chromatin by histone modifications. Cell Res. 21, 381–395 10.1038/cr.2011.22 21321607 PMC3193420

[B4] Blandin S. A. Marois E. Levashina E. A. (2008). Antimalarial responses in *Anopheles gambiae*: from a complement-like protein to a complement-like pathway. Cell Host Microbe 3, 364–374 10.1016/j.chom.2008.05.007 18541213

[B5] Bonasio R. Tu S. Reinberg D. (2010). Molecular signals of epigenetic states. Science 330, 612–616 10.1126/science.1191078 21030644 PMC3772643

[B6] Bonduriansky R. Day T. (2009). Non-genetic inheritance and its evolutionary implications. Annu. Rev. Ecol. Evol. Syst. 40, 103–125 10.1146/annurev.ecolsys.39.110707.173441

[B7] Bowman S. Simon M. Deaton A. Tolstorukov M. Borowsky M. Kingston R. (2013). Multiplexed Illumina sequencing libraries from picogram quantities of DNA. BMC Genomics 14:466 10.1186/1471-2164-14-466 23837789 PMC3711846

[B8] Brown E. J. Bachtrog D. (in press). The chromatin landscape of Drosophila: comparisons between species, sexes, and chromosomes. Genome Res. 10.1101/gr.172155.114 24840603 PMC4079968

[B9] Cheng C. Yan K.-K. Yip K. Y. Rozowsky J. Alexander R. Shou C. (2011). A statistical framework for modeling gene expression using chromatin features and application to modENCODE datasets. Genome Biol. 12:R15 10.1186/gb-2011-12-2-r15 21324173 PMC3188797

[B10] Creyghton M. P. Cheng A. W. Welstead G. G. Kooistra T. Carey B. W. Steine E. J. (2010). Histone H3K27ac separates active from poised enhancers and predicts developmental state. Proc. Natl. Acad. Sci. U.S.A. 107, 21931–21936 10.1073/pnas.1016071107 21106759 PMC3003124

[B11] Dana A. N. Hong Y. S. Kern M. K. Hillenmeyer M. E. Harker B. W. Lobo N. F. (2005). Gene expression patterns associated with blood-feeding in the malaria mosquito Anopheles gambiae. BMC Genomics 6:5 10.1186/1471-2164-6-5 15651988 PMC546002

[B12] Ernst J. Kellis M. (2010). Discovery and characterization of chromatin states for systematic annotation of the human genome. Nat. Biotechnol. 28, 817–825 10.1038/nbt.1662 20657582 PMC2919626

[B13] Feng J. Liu T. Qin B. Zhang Y. Liu X. S. (2012). Identifying ChIP-seq enrichment using MACS. Nat. Protoc. 7, 1728–1740 10.1038/nprot.2012.101 22936215 PMC3868217

[B14] Filion G. J. Van Bemmel J. G. Braunschweig U. Talhout W. Kind J. Ward L. D. (2010). Systematic protein location mapping reveals five principal chromatin types in *Drosophila* cells. Cell 143, 212–224 10.1016/j.cell.2010.09.009 20888037 PMC3119929

[B15] Gómez-Díaz E. Jordà M. Peinado M. A. Rivero A. (2012). Epigenetics of host-pathogen interactions: the road ahead and the road behind. PLoS Pathog. 8:e1003007 10.1371/journal.ppat.1003007 23209403 PMC3510240

[B16] Hoeijmakers W. A. Stunnenberg H. G. Bartfai R. (2012). Placing the *Plasmodium falciparum* epigenome on the map. Trends Parasitol. 28, 486–495 10.1016/j.pt.2012.08.006 22999479

[B17] Holt R. A. Subramanian G. M. Halpern A. Sutton G. G. Charlab R. Nusskern D. R. (2002). The genome sequence of the malaria mosquito *Anopheles gambiae*. Science 298, 129–149 10.1126/science.1076181 12364791

[B18] Hou C. Li L. Qin Z. S. Corces V. G. (2012). Gene density, transcription, and insulators contribute to the partition of the Drosophila genome into physical domains. Mol. Cell 48, 471–484 10.1016/j.molcel.2012.08.031 23041285 PMC3496039

[B19] Kellner W. A. Ramos E. Van Bortle K. Takenaka N. Corces V. G. (2012). Genome-wide phosphoacetylation of histone H3 at Drosophila enhancers and promoters. Genome Res. 22, 1081–1088 10.1101/gr.136929.111 22508764 PMC3371715

[B20] Kharchenko P. V. Alekseyenko A. A. Schwartz Y. B. Minoda A. Riddle N. C. Ernst J. (2011). Comprehensive analysis of the chromatin landscape in *Drosophila melanogaster*. Nature 471, 480–485 10.1038/nature09725 21179089 PMC3109908

[B21] Koutsos A. C. Blass C. Meister S. Schmidt S. Maccallum R. M. Soares M. B. (2007). Life cycle transcriptome of the malaria mosquito *Anopheles gambiae* and comparison with the fruitfly *Drosophila melanogaster*. Proc. Natl. Acad. Sci. U.S.A. 104, 11304–11309 10.1073/pnas.0703988104 17563388 PMC2040894

[B22] Kouzarides T. (2007). Chromatin modifications and their function. Cell 128, 693–705 10.1016/j.cell.2007.02.005 17320507

[B23] Lambrechts L. (2010). Dissecting the genetic architecture of host–pathogen specificity. PLoS Pathog. 6:e1001019 10.1371/journal.ppat.1001019 20700450 PMC2916863

[B24] Landt S. G. Marinov G. K. Kundaje A. Kheradpour P. Pauli F. Batzoglou S. (2012). ChIP-seq guidelines and practices of the ENCODE and modENCODE consortia. Genome Res. 22, 1813–1831 10.1101/gr.136184.111 22955991 PMC3431496

[B25] Langmead B. Salzberg S. L. (2012). Fast gapped-read alignment with Bowtie 2. Nat. Methods 9, 357–359 10.1038/nmeth.1923 22388286 PMC3322381

[B26] Maccallum R. M. Redmond S. N. Christophides G. K. (2011). An expression map for *Anopheles gambiae*. BMC Genomics 12:620 10.1186/1471-2164-12-620 22185628 PMC3341590

[B27] Marinotti O. Nguyen Q. K. Calvo E. James A. A. Ribeiro J. M. (2005). Microarray analysis of genes showing variable expression following a blood meal in *Anopheles gambiae*. Insect Mol. Biol. 14, 365–373 10.1111/j.1365-2583.2005.00567.x 16033430

[B28] Negre N. Brown C. D. Ma L. Bristow C. A. Miller S. W. Wagner U. (2011). A cis-regulatory map of the Drosophila genome. Nature 471, 527–531 10.1038/nature09990 21430782 PMC3179250

[B29] Pauler F. M. Sloane M. A. Huang R. Regha K. Koerner M. V. Tamir I. (2009). H3K27me3 forms BLOCs over silent genes and intergenic regions and specifies a histone banding pattern on a mouse autosomal chromosome. Genome Res. 19, 221–233 10.1101/gr.080861.108 19047520 PMC2652204

[B30] Pitts R. J. Rinker D. C. Jones P. L. Rokas A. Zwiebel L. J. (2011). Transcriptome profiling of chemosensory appendages in the malaria vector *Anopheles gambiae* reveals tissue-and sex-specific signatures of odor coding. BMC Genomics 12:271 10.1186/1471-2164-12-271 21619637 PMC3126782

[B31] Quinlan A. R. Hall I. M. (2010). BEDTools: a flexible suite of utilities for comparing genomic features. Bioinformatics 26, 841–842 10.1093/bioinformatics/btq033 20110278 PMC2832824

[B32] Ramírez F. Dündar F. Diehl S. Grüning B. A. Manke T. (in press). DeepTools: a flexible platform for exploring deep-sequencing data. Nucleic Acids Res. 10.1093/nar/gku365 24799436 PMC4086134

[B33] Riddle N. C. Minoda A. Kharchenko P. V. Alekseyenko A. A. Schwartz Y. B. Tolstorukov M. Y. (2011). Plasticity in patterns of histone modifications and chromosomal proteins in Drosophila heterochromatin. Genome Res. 21, 147–163 10.1101/gr.110098.110 21177972 PMC3032919

[B34] Roberts A. Pimentel H. Trapnell C. Pachter L. (2011). Identification of novel transcripts in annotated genomes using RNA-Seq. Bioinformatics 27, 2325–2329 10.1093/bioinformatics/btr355 21697122

[B35] Robertson G. Hirst M. Bainbridge M. Bilenky M. Zhao Y. Zeng T. (2007). Genome-wide profiles of STAT1 DNA association using chromatin immunoprecipitation and massively parallel sequencing. Nat. Methods 4, 651–657 10.1038/nmeth1068 17558387

[B36] Roy S. Ernst J. Kharchenko P. V. Kheradpour P. Negre N. Eaton M. L. (2010). Identification of functional elements and regulatory circuits by Drosophila modENCODE. Science 330, 1787–1797 10.1126/science.1198374 21177974 PMC3192495

[B37] Schuettengruber B. Ganapathi M. Leblanc B. Portoso M. Jaschek R. Tolhuis B. (2009). Functional anatomy of polycomb and trithorax chromatin landscapes in Drosophila embryos. PLoS Biol. 7:e13 10.1371/journal.pbio.1000013 19143474 PMC2621266

[B38] Schwartz Y. B. Kahn T. G. Nix D. A. Li X.-Y. Bourgon R. Biggin M. (2006). Genome-wide analysis of Polycomb targets in *Drosophila melanogaster*. Nat. Genet. 38, 700–705 10.1038/ng1817 16732288

[B39] Severson D. W. Behura S. K. (2012). Mosquito genomics: progress and challenges. Annu. Rev. Entomol. 57, 143–166 10.1146/annurev-ento-120710-100651 21942845

[B40] Tolhuis B. De Wit E. Muijrers I. Teunissen H. Talhout W. Van Steensel B. (2006). Genome-wide profiling of PRC1 and PRC2 Polycomb chromatin binding in *Drosophila melanogaster*. Nat. Genet. 38, 694–699 10.1038/ng1792 16628213

[B41] Trapnell C. Pachter L. Salzberg S. L. (2009). TopHat: discovering splice junctions with RNA-Seq. Bioinformatics 25, 1105–1111 10.1093/bioinformatics/btp120 19289445 PMC2672628

[B42] Trapnell C. Roberts A. Goff L. Pertea G. Kim D. Kelley D. R. (2012). Differential gene and transcript expression analysis of RNA-seq experiments with TopHat and Cufflinks. Nat. Protoc. 7, 562–578 10.1038/nprot.2012.016 22383036 PMC3334321

[B43] Van Bortle K. Ramos E. Takenaka N. Yang J. Wahi J. E. Corces V. G. (2012). Drosophila CTCF tandemly aligns with other insulator proteins at the borders of H3K27me3 domains. Genome Res. 22, 2176–2187 10.1101/gr.136788.111 22722341 PMC3483547

[B44] Vannini L. Augustine Dunn W. Reed T. W. Willis J. H. (2014). Changes in transcript abundance for cuticular proteins and other genes three hours after a blood meal in *Anopheles gambiae*. Insect Biochem. Mol. Biol. 44, 33–43 10.1016/j.ibmb.2013.11.002 24269292 PMC3970321

[B45] Visel A. Blow M. J. Li Z. Zhang T. Akiyama J. A. Holt A. (2009). ChIP-seq accurately predicts tissue-specific activity of enhancers. Nature 457, 854–858 10.1038/nature07730 19212405 PMC2745234

[B46] Voigt P. Tee W.-W. Reinberg D. (2013). A double take on bivalent promoters. Genes Dev. 27, 1318–1338 10.1101/gad.219626.113 23788621 PMC3701188

[B47] Wang D. Bodovitz S. (2010). Single cell analysis: the new frontier in ‘omics’. Trends Biotechnol. 28, 281–290 10.1016/j.tibtech.2010.03.002 20434785 PMC2876223

[B48] Who. (2013). World Malaria Report: 2013. Geneva: World Health Organization

[B49] Xu S. Grullon S. Ge K. Peng W. (2014). Spatial Clustering for Identification of ChIP-Enriched Regions (SICER) to map regions of histone methylation patterns in embryonic stem cells, in Stem Cell Transcriptional Networks, ed Kidder B. L. (New York, NY: Springer), 97–111 10.1007/978-1-4939-0512-6_5 PMC4152844 24743992

[B50] Yin H. Sweeney S. Raha D. Snyder M. Lin H. (2011). A high-resolution whole-genome map of key chromatin modifications in the adult *Drosophila melanogaster*. PLoS Genet. 7:e1002380 10.1371/journal.pgen.1002380 22194694 PMC3240582

[B51] Young M. D. Willson T. A. Wakefield M. J. Trounson E. Hilton D. J. Blewitt M. E. (2011). ChIP-seq analysis reveals distinct H3K27me3 profiles that correlate with transcriptional activity. Nucleic Acids Res. 39, 7415–7427 10.1093/nar/gkr416 21652639 PMC3177187

[B52] Zang C. Schones D. E. Zeng C. Cui K. Zhao K. Peng W. (2009). A clustering approach for identification of enriched domains from histone modification ChIP-Seq data. Bioinformatics 25, 1952–1958 10.1093/bioinformatics/btp340 19505939 PMC2732366

[B53] Zhang Y. Liu T. Meyer C. A. Eeckhoute J. Johnson D. S. Bernstein B. E. (2008). Model-based analysis of ChIP-Seq (MACS). Genome Biol. 9:R137 10.1186/gb-2008-9-9-r137 18798982 PMC2592715

[B54] Zhu Y. Sun L. Chen Z. Whitaker J. W. Wang T. Wang W. (2013). Predicting enhancer transcription and activity from chromatin modifications. Nucleic Acids Res. 41, 10032–10043 10.1093/nar/gkt826 24038352 PMC3905895

