# Supplementary figures and images for: Insights into the epigenomic landscape of the human malaria vector Anopheles gambiae
Source: Front Genet. 2014 Aug 15;5:277. doi: 10.3389/fgene.2014.00277 (PMC4133732; doi:10.3389/fgene.2014.00277)

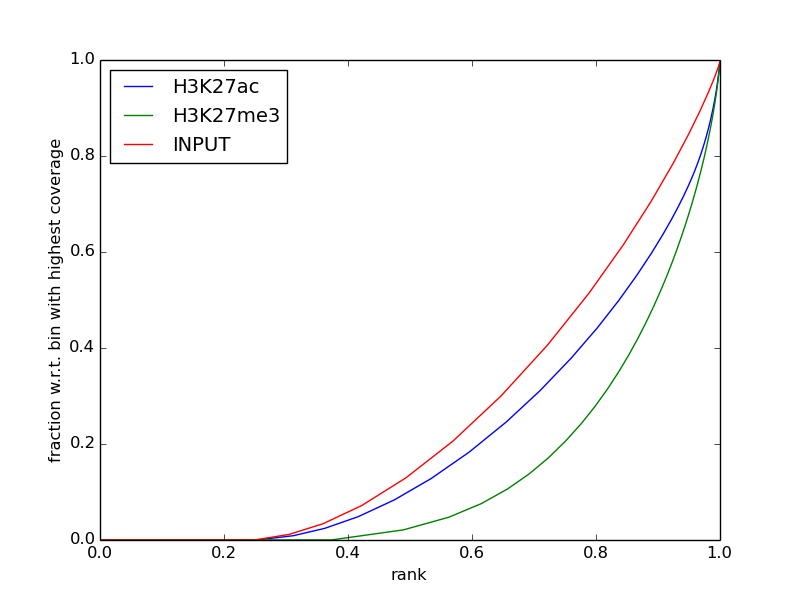

Supplement: Figure S1 — Fingerprint plot that shows the distribution of reads, as the cumulative sum of read counts in 10 bp window bins, for H3K27ac, H3K27me3, and input samples. A tight diagonal is expected when reads are equally distributed across the genome, as is the case for the input, whereas the curve becomes more pronounced as the degree of enrichment increases and is more localized in the ChIPs relative to the control sample. [file Image1.TIF]

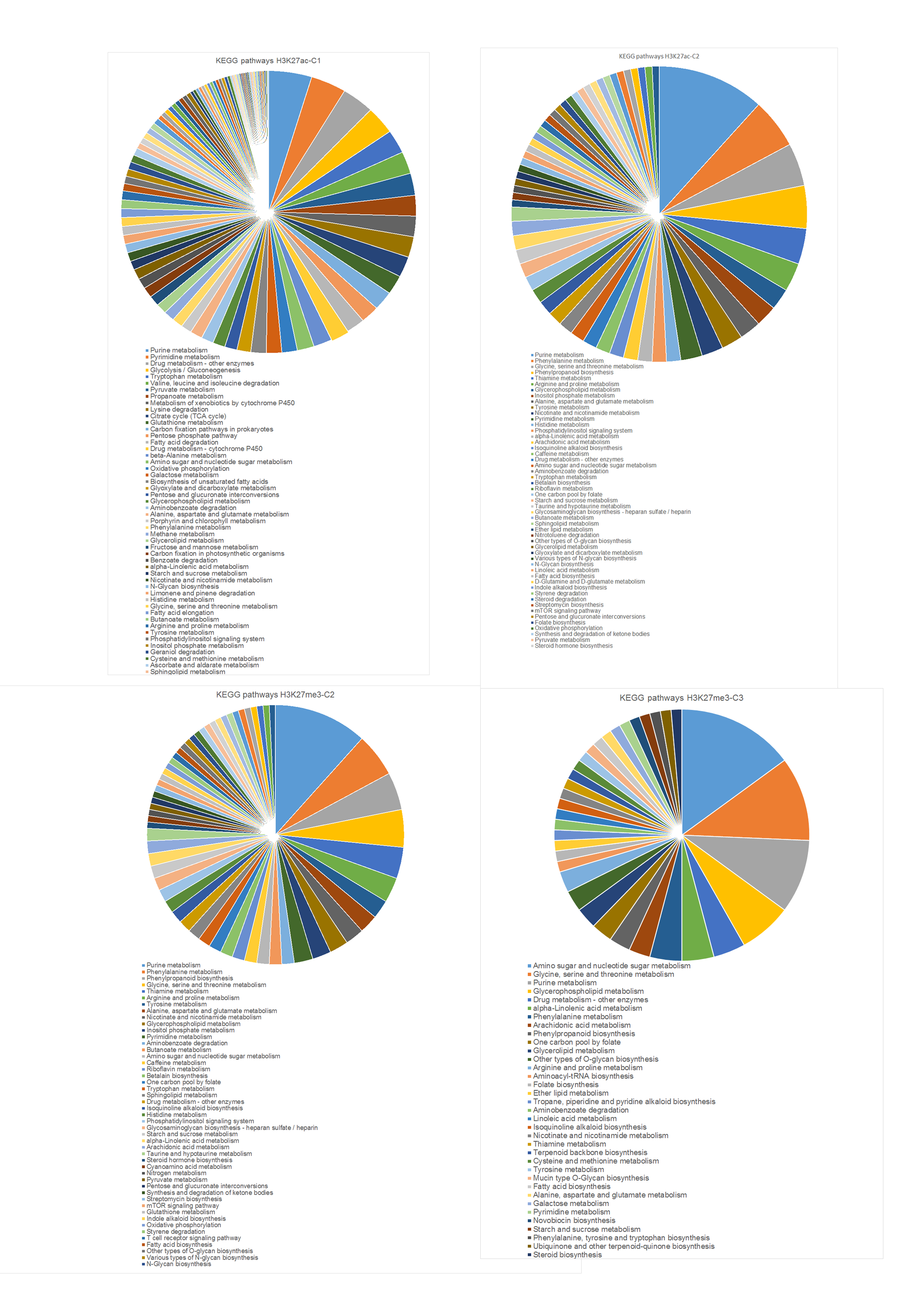

Supplement: Figure S2 — Piecharts that show the number of sequences assigned to KEGG pathways for each histone modification gene cluster (see text, Figure 2). [file Image2.TIF]
