# Supplementary material for: Insights into the epigenomic landscape of the human malaria vector Anopheles gambiae
Source: Front Genet. 2014 Aug 15;5:277. doi: 10.3389/fgene.2014.00277 (PMC4133732; doi:10.3389/fgene.2014.00277)
Supplement: Supplementary file 3 [file DataSheet1.DOCX]

Table S1. List of primers used in the qPCR-ChIP validation

| **Gene** | **VectorBase ID** | **Primer sequence (5’ to 3’)** |
| --- | --- | --- |
| *Act5C* | AGAP000651 | GGTTTTAAGCACGAAAAACTGG |
| *Act5C* | AGAP000651 | CGATCCGGAGAGGTCCTAC |
| *TER94* | AGAP005630 | TCGATTCGACTCAAGCTTCC |
| *TER94* | AGAP005630 | AGTCATCGTCATCGCTGCTT |
